# Undifferentiated Connective Tissue Disease With Isolated Diaphragmatic Dysfunction

**DOI:** 10.7759/cureus.40515

**Published:** 2023-06-16

**Authors:** Richa Purohit, Ravi Shahu Khal, Gizem Gokalp, Rajan Sambandan, Neha Bhanusali

**Affiliations:** 1 Department of Medicine, Concentra Urgent Care, Orlando, USA; 2 Department of Rheumatology, University of Central Florida Hospital Corporation of America (HCA) Healthcare Graduate Medical Education (GME), Orlando, USA; 3 Department of Medicine, University of Central Florida Hospital Corporation of America (HCA) Healthcare Graduate Medical Education (GME), Orlando, USA; 4 Department of Medicine, University of Central Florida College of Medicine, Orlando, USA; 5 Department of Rheumatology, University of Central Florida College of Medicine, Orlando, USA

**Keywords:** pulmonary function test, ivig, diaphragmatic dysfunction, dyspnea, undifferentiated connective tissue disease

## Abstract

Undifferentiated connective tissue disease (UCTD) is a rare autoimmune disorder with a prevalence of about two people per 100,000 people per year. Patients present with the features of different connective tissue diseases, including systemic lupus erythematosus, systemic sclerosis, polymyositis, and rheumatoid arthritis, with some positivity in serological markers that is insufficient to fulfill the criteria of any recognized connective tissue disorder. Pulmonary involvement is usually subacute and pleomorphic, which can cause a delay in the diagnosis. A few cases of UCTD involving an isolated diaphragm in the pulmonary system have been reported. We report a case of a 48-year-old female who initially presented with various nonspecific symptoms, including fatigue, polyarthralgia, dry mouth, and Raynaud’s phenomenon. Subsequently, she developed significant dyspnea and orthopnea. Laboratory, immunology, and imaging workups were negative for any specific diagnosis. Pulmonary function tests showed severely low maximum inspiratory pressure (MEP) and maximum expiratory pressure, suggesting diaphragmatic dysfunction. A diagnosis of UCTD was considered, and she was treated with hydroxychloroquine and intravenous immunoglobulin (IVIG), which improved her respiratory symptoms and pulmonary function tests.

## Introduction

Undifferentiated connective tissue disease (UCTD) is often challenging to diagnose and manage, given the lack of specific guidelines. Patients present with clinical manifestations of rheumatological diseases with some positivity in serological markers that is insufficient to fulfill the criteria of any recognized connective tissue disorder [1]. The signs and symptoms of UCTD can be variable. Lung involvement in the form of interstitial lung disease with a nonspecific interstitial pneumonia (NSIP) pattern has been reported in association with UCTD [2]. Diaphragm dysfunction has not been described in UCTD per se. However, it is commonly associated with several rheumatological conditions, including inflammatory myositis [3]. Diaphragmatic dysfunction often presents with symptoms of dyspnea, intolerance to exercise, sleep disturbances, and hypersomnia. In our case, we discuss a unique presentation of UCTD with shortness of breath and excessive yawning.

## Case presentation

A 48-year-old woman presented with complaints of polyarthralgia, myalgia, tingling, numbness in the upper extremities, occasional dry eyes and dry mouth, and occasional dysphagia for both solid and liquid diets. All these symptoms started around the spring of 2015. The review of systems was also positive for Raynaud’s phenomenon. No synovitis was noticed on the musculoskeletal examination, and no deficits were detected on the neurological examination. Her skin was also normal overall. The only positive finding on examination was dilated capillaries and microhemorrhages in her right-hand fourth and fifth nail folds on capillaroscopy. She was initiated on hydroxychloroquine, which improved her joint pain.

However, around the fall of 2017, despite being on hydroxychloroquine, she developed progressive dyspnea, excessive yawning, and dysphagia of solids and liquids that usually occurred in the evening. The shortness of breath was due to minimal exertion, and she had also started developing orthopnea. She also described excessive yawning throughout the day and generalized fatigue.

On lab workup, general labs, including complete blood count (CBC), complete metabolic profile (CMP), thyroid function panel, hepatitis panel, syphilis, celiac disease, and sarcoidosis, were all normal. The erythrocyte sedimentation rate (ESR) was 65, and the anti-nuclear antibody (ANA) titer was 1:160. In the Mayo Clinic myositis panel, the anti-Ku antibody was weakly positive. She was evaluated for myasthenia gravis, and the acetylcholine receptor antibodies were found to be negative, making myasthenia gravis a less probable diagnosis for her symptoms. Her initial Sjogren’s panel also showed weak positive anti-SSA (1.7, n<1). However, on repeat tests one month later, the Sjogren’s panel, including the anti-SSA/SSB panel and the myositis panel, were both negative. Her muscle enzymes, creatine phosphokinase (CPK) and aldolase, were within normal limits. She also tested negative for several other antibody panels, such as anti-dsDNA antibody, anti-Sm/RNP, SSB, SCL70, anti-centromere, anti-cyclic citrullinated peptide (CCP), anticardiolipin antibody, beta-2 glycoprotein antibody, paraneoplastic antibody panel, anti-phospholipid antibody panel, and anti-nuclear cytoplasmic antibodies. Genetic testing for type 1 and type 2 myotonic dystrophy and limb-girdle muscular dystrophy was negative for pathogenic mutations.

An extensive imaging workup was done for dyspnea. A high-resolution computed tomography (HRCT) chest and ventilation-perfusion (VQ) scan did not reveal any pulmonary pathology. The cardiac workup, including an echocardiogram and exercise stress test, was within normal limits. The computed tomography of the abdomen/pelvis did not reveal any abnormalities. Bilateral hand X-rays did not reveal any abnormalities. Nerve conduction velocity (NCV)/electromyography (EMG) studies of the diaphragm and the bilateral upper and lower extremities were normal. The diaphragm was also evaluated by fluoroscopy and ultrasound; neither revealed any abnormal movement or pattern. The patient also underwent a lip biopsy that did not suggest evidence of Sjogren’s syndrome. A right quadriceps femoris biopsy was done that did not reveal any evidence of inflammatory myopathy. The swallow study showed only transient laryngeal penetration and no other abnormalities. MRIs of the brain and cervical spine were also negative for any intracranial or spinal abnormalities. Pulmonology was consulted, and the patient had pulmonary function tests (PFTs) completed. It indicated a severely low maximum inspiratory pressure (MIP) and maximum expiratory pressure (MEP), consistent with respiratory muscle weakness.

**Figure 1:**
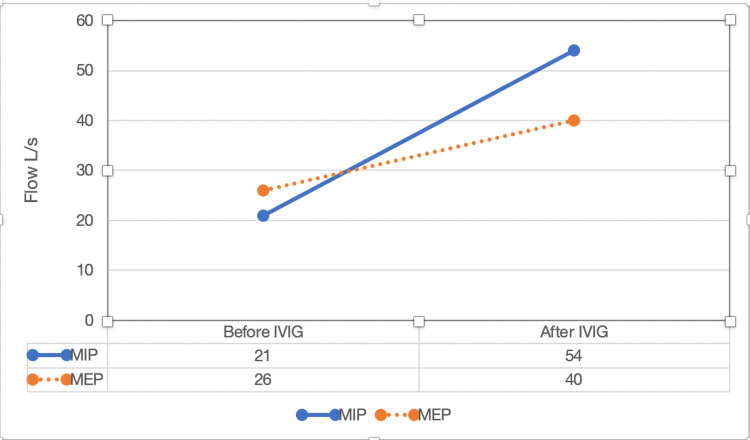
Change in maximum inspiratory pressure and maximum expiratory pressure after IVIG treatment. Reference ranges, respectively, are >81 L/s and >146 L/s.

Persistent respiratory symptoms despite being on hydroxychloroquine and tapering steroids prompted the addition of intravenous immunoglobulin (IVIG) treatment for a presumptive diagnosis of UCTD with isolated diaphragmatic dysfunction. The patient started showing improvement with the second dose of IVIG. Her shortness of breath and yawning clinically improved, and the patient could resume activities of daily living. Additionally, there was an objective improvement in the PFTS (Figure 1).

The MIP improved from 21 to 54 (normal level is >81 L/s), and the MEP improved from 26 to 40 (normal level is >146 L/s). The patient had some insurance issues, so the IVIG therapy had to be put on hold twice, once for around six to seven weeks and the second time for approximately three weeks. Both times, the patient’s symptoms started returning while she was off IVIG and would improve when she resumed the treatment.

The patient’s response to IVIG, with significant improvement in her symptoms and pulmonary functions, is highly suggestive of an underlying immunologic basis. Given the array of symptoms with elevated ANA and all other specific tests being negative, the diagnosis of UCTD with isolated diaphragmatic dysfunction was determined.

## Discussion

Diaphragmatic dysfunction can result from various causes, such as disorders of spinal cord motor neurons, neuromuscular diseases, and muscle diseases like inflammatory myopathies. IVIG has been shown to be effective in treating diaphragmatic dysfunction related to inflammatory myositis, myasthenia gravis, and Guillain-Barre syndrome [4]. In our case, neuromuscular testing and myasthenia gravis antibodies were negative, ruling out neuromuscular diseases. Similarly, the brain and cervical spine MRIs were negative for any intracranial or spinal cord pathology.

Diaphragm weakness is frequent and probably overlooked in inflammatory myopathies. A study of 23 consecutive patients (12 with polymyositis, five with dermatomyositis, and six with inclusion body myositis) found 18 patients (78%) diagnosed with diaphragmatic weakness [4]. Interestingly, our case did not have the typical findings of inflammatory myopathy, as evidenced by normal muscle enzymes, normal muscle strength on the exam, and a negative muscle biopsy. Features of UCTD and significant clinical and PFT responses to IVIG suggest an underlying immunological basis for diaphragmatic dysfunction.

The literature review did not yield any documented evidence of yawning or diaphragmatic dysfunction related to UCTD or any beneficial treatment effect of IVIG. One case report describes acute respiratory failure due to diaphragmatic weakness related to polymyositis successfully treated with IVIG and corticosteroids [5]. Similarly, there is another case report of bilateral diaphragmatic dysfunction related to inclusion body myositis managed with inspiratory muscle training and an aerobic exercise training program [6]. In the literature, excessive yawning has been associated with different neurological conditions, including multiple sclerosis, amyotrophic lateral sclerosis, stroke, and seizures [7]. In our literature review, the correlation between diaphragmatic dysfunction and yawning was limited. One review suggested neuromuscular disorders had decreased compliance with limited diaphragm movement, which increased spontaneous yawns [8].

## Conclusions

Pulmonary manifestations in UCTD are common. However, isolated diaphragmatic dysfunction can be challenging for clinicians, especially when the extensive workup is negative. A diaphragm biopsy confirms the diagnosis but is invasive. Diaphragmatic fluoroscopy and neuromuscular velocity studies can help support the diagnosis. Though steroids and other immunosuppressive agents have been shown to improve other rheumatological manifestations of UCTD, our case indicates that IVIG can also effectively treat diaphragmatic dysfunction associated with UCTD.
